# Clinical Study of Wrist Arthroscopy Combined with Oblique Ulnar Shortening Osteotomy in the Treatment of Ulnar Impaction Syndrome

**DOI:** 10.1111/os.13428

**Published:** 2022-09-30

**Authors:** Chengyin Lu, Pengtao Wang, Laifu Zhang, Jiahe Dong, Hailong Zhang, Lei Yang, Xiaohui Wang, Hui Xiong

**Affiliations:** ^1^ Department of Graduate School Hunan University of Chinese Medicine Changsha China; ^2^ Department of Sports Medicine Luoyang Orthopedic‐Traumatological Hospital of Henan Province (Henan Provincial Orthopedic Hospital) Luoyang China; ^3^ Department of Graduate School Henan University of Chinese Medicine Zhengzhou China

**Keywords:** arthroscopy, osteotomy, triangular fibrocartilage, ulnar, wrist

## Abstract

**Objective:**

To explore the clinical effects of wrist arthroscopy combined with oblique ulnar shortening osteotomy in the treatment of ulnar impaction syndrome.

**Methods:**

This was a retrospective study of 60 patients with ulnar impaction syndrome who were admitted to our department from January 2016 to December 2019. According to different surgical methods, they were divided into an observation group and a control group, with 30 cases in each group. The control group was treated with oblique ulnar shortening osteotomy, and the observation group used wrist arthroscopy based on the control group. The two groups of patients were compared in terms of wrist function before and 12 months after surgery. We compared the Disabilities of the Arm, Shoulder, and Hand Score (DASH Score), Patient‐Rated Wrist Evaluation Score (PRWE Score), Visual Analog Score (VAS), and ulnar variation between the two groups at 12 months after surgery. The excellent and good rates by Mayo wrist score were compared between the two groups at the last follow‐up.

**Results:**

All patients were followed up for 12–36 months, with an average of 14.5 months. Bone union was achieved at the oblique osteotomy of the ulna, with an average healing time of 13.6 weeks. The observation group was examined by wrist arthroscopy, and the triangular fibrocartilage complex (TFCC) Palmer classification was confirmed in three cases of type IIA, seven cases of type IIB, 14 cases of type IIC, and six cases of type IID. Compared with before surgery, the grip strength, flexion‐extension, ulnar radial deflection, and forearm rotation of the two groups of patients were significantly improved at 12 months after surgery. The DASH score, PRWE score, and VAS score of the observation group were better than those of the control group, and the difference in ulnar shortening length was not statistically significant between the two groups. The excellent and good rates of the observation group (93.3%) were better than those of the control group (87.5%) at the last follow‐up, and the difference was statistically significant.

**Conclusion:**

Compared with oblique ulnar shortening osteotomy alone, combined wrist arthroscopy can better reduce the pain of patients with ulnar impaction syndrome, restore wrist function, and improve the excellent and good rates. Therefore, it is clinically worthy of promotion.

## Graphical abstract

Wrist arthroscopy combined with oblique ulnar shortening osteotomy in the treatment of ulnar impaction syndrome can restore the positive ulnar variation while repairing soft tissue damage such as TFCC, which can better relieve the pain of patients, improve the rate of excellent and good, and increase the satisfaction of treatment.

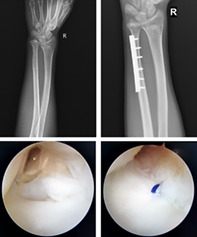

## Introduction

Ulnar impaction syndrome (UIS) is a syndrome caused by the positive variance of the ulna due to various factors^1^, which causes the ulna to continuously hit the wrist. This results in wear of the triangular fibrocartilage complex (TFCC), injury of the scapholunate ligament, lunotriquetral ligament and chondromalacia of the lunate and triquetrum, leading to pain on the ulnar side of the wrist, limited movement, and decreased grip strength. It is one of the common wrist diseases in clinics^2^. The causes of UIS include malunion of radius fractures, congenital positive ulnar variation, and chronic strain leading to the long‐term impact of the ulnar wrist joint^3^. Conservative treatment can only temporarily relieve wrist pain but cannot be treated thoroughly. The key to the treatment of this disease is to accurately and effectively reduce the ulnar positive variation and restore the wrist's anatomical structure^4^. Therefore, surgery is still the main treatment for UIS. At present, the clinical surgical methods include ulnar shortening osteotomy (USO)^5^, arthroscopic wafer surgery^6^, and Sauve‐Kapandji surgery^7^. Among them, USO is the most commonly used in the clinic. The principle of USO is to reduce the axial stress of the ulna to the wrist by correcting the positive variation of the ulna to relieve the pain symptoms. Because the operation is located outside the wrist joint, it does not damage the starting and ending points of the soft tissue around the wrist joint, so it can better maintain the mechanical integrity of the lower radioulnar joint^8^. The osteotomy methods of USO include transverse osteotomy, oblique osteotomy, V‐shaped osteotomy, trapezoidal osteotomy, etc. Transverse osteotomy is relatively simple, but the contact area of both ends of the fracture is small, and is prone to rotation displacement, so the fracture healing time is long. The contact area of both ends of V‐shaped and trapezoidal osteotomy fractures is large, and the anti‐rotation force is enhanced, but the operation is more complicated, the operation time is long, and the two ends of the fracture are prone to malalignment^9^. However, oblique osteotomy is easy to perform, with a large contact area and strong anti‐rotation force, so it is widely used in the clinic^10^. Terzis *et al*.^11^ treated 17 patients with UIS with oblique USO and followed them up. The results showed that all patients had significant pain relief, wrist function recovered well, and patient satisfaction was high.

Although oblique USO has achieved good results in the treatment of UIS, it was reported that some patients still had wrist pain after oblique USO^12^. Rodriguez and Eglseder^13^ treated 16 patients with UIS using an oblique USO, and 14 of the 16 patients were found to have persistent occasional pain during certain activities at follow‐up. Chan's^14^ retrospective study also reported that 1.6% of patients had residual ulnar‐sided pain of unknown cause after USO. Koh *et al*.^15^ thinks that it may be related to the wear or tear of TFCC. For these patients, only oblique USO with no repair of TFCC may lead to poor postoperative results^16^. In recent years, with the development of wrist arthroscopy, the treatment effect of UIS has been further improved. Wrist arthroscopy can not only accurately diagnose the degree and classification of TFCC damage but can also perform corresponding treatments in a minimally invasive manner^17^. However, there are still few reports on the treatment of UIS with wrist arthroscopy combined with oblique USO.

Therefore, the purpose of this study was (i) to compare the clinical efficacy of wrist arthroscopy combined with oblique USO and oblique USO alone in the treatment of UIS and (ii) to summarize the key surgical points and precautions for the treatment of UIS by the wrist arthroscopic technique combined with oblique USO.

## Methods

### Inclusion and Exclusion Criteria

The diagnostic criteria for UIS were as follows: (i) patients with ulnar pain, increased pain, and limited activity with rotation, deviation, or compression; (ii) a positive stress test on the ulnar side; (iii) a positive variance in the ulna and a millimeter of ulnar positive variance >2 mm on X‐ray examination of the wrist; and (iv) MRI showing signal changes in the lunate, triquetrum, or TFCC.

The criteria for inclusion were as follows: (i) patients with UIS were diagnosed; (ii) MRI showed TFCC, scapholunate ligament, or lunotriquetral ligament injury, or cystic changes of the lunate and triquetrum.

The criteria for exclusion were as follows: (i) patients who chose conservative treatment; (ii) patients with abnormal preoperative blood, urine, fecal, liver, and kidney function, coagulation function, and other test results; (iii) patients who had serious medical diseases and could not tolerate surgery and anesthesia; and (iv) incomplete follow‐up data.

All patients in this study signed informed consent forms.

### General Information

A total of 60 patients were included in this study. Depending on the surgical methods, we chose wrist arthroscopy combined with oblique USO for the observation group and oblique USO alone for the control group. In the observation group, there were 30 cases, 12 males and 18 females, aged from 23 to 68 years, with an average of 43.4 years. There were 11 cases on the left side and 19 cases on the right side. The causes were as follows: old distal radius fracture secondary ulnar positive variation in eight cases, wrist joint repeated labour for a long time due to work or life in 16 cases, and no obvious inducement in six cases. The course of disease was 8–60 months, with an average of 15.8 months. In the control group, there were 30 cases, 13 males and 17 females, aged from 18 to 72 years, with an average of 41.6 years. There were 13 cases on the left side and 17 cases on the right side. The causes were old distal radius fracture secondary ulnar positive variation in six cases, wrist joint repeated labour for a long time due to work or life in 17 cases, and no obvious inducement in seven cases. The course of disease was 6–48 months, with an average of 16.4 months. The two groups of patients had no statistically significant differences in general information, such as sex, age, affected side, course of disease, and cause of treatment, and they were comparable (*p* > 0.05). This retrospective study was approved by the ethics committee of Luoyang Orthopedic‐Traumatological Hospital of Henan Province (Henan Provincial Orthopedic Hospital) (no. KX2017‐002‐02).

## Surgical Methods

### Oblique Ulnar Shortening Osteotomy (Oblique USO)

The control group was treated with oblique USO.

Step 1: The patient was placed in a supine position, and a tourniquet was used to stop the bleeding. Starting from the ulnar neck, a longitudinal incision was made on the ulnar side of the distal forearm to expose the subperiosteal dorsal side of the ulna, a piece of six‐hole or seven‐hole dynamic compression plate was placed on the dorsal side of the distal ulna, and the distal end of the plate was approximately at the proximal position of the ulnar neck and the sigmoid notch.
Step 2: The two screws were tightened at the distal end of the plate. The position of the ulna osteotomy was generally between the third and fourth holes of the plate. A marker was used to mark the position of the osteotomy.
Step 3: The second screw was removed, the plate was rotated, and the osteotomy length was determined according to the positive ulnar variation value measured before the operation. Generally, the osteotomy length was 1–2 mm longer than the positive variation value. Both ends of the osteotomy were inserted into two parallel K‐wires. The K‐wires were used as a reduction marker and a tool to assist with the osteotomy. A swing saw was used to perform osteotomy within two K‐wires. After the osteotomy was completed, the osteotomy block was removed, and the ulna osteotomy was reset. Then, a K‐wire was inserted obliquely into the ulna to fix both ends of the osteotomy, the two assisted K‐wires were removed, and the plate was rotated to determine the position on the ulna.
Step 4: The first screw was replaced and tightened and then the second screw was tightened. The proximal bone block was fixed with bone clamps, and dynamic compression technology was used to tighten the remaining screws in turn to remove the oblique gram that fixes both ends of the osteotomy.
Step 5: After fluoroscopic confirmation of the alignment and ulna length, the incision was sutured layer by layer, and the drainage strip was placed.

### Wrist Arthroscopy Technique to Explore and Repair Wrist Joints

Based on the control group, the observation group used wrist arthroscopy to explore and repair the wrist joints.

Step 1: Wrist arthroscopy and traction towers were installed, and finger cuffs were used to traction two or three fingers to suspend and fix the affected limb. Lister nodules were marked, and the 3/4 approach was located. Then, 5–10 mL of saline was injected from the 3/4 approach, and the 3/4, 6R, 4/5, and 6U approaches were established for wrist arthroscopy.
Step 2: The scapholunar ligament, radioscaphcapitate ligament, radiolunate ligament, and other ligaments and TFCC conditions were observed. TFCC injuries were classified using the Palmer classification and repaired accordingly.
Step 3: Debridement and chondroplasty were used to repair the TFCC type IIA and IIB injuries, and the “outside‐in” technique loop method was used to fix the types IIC and IID.

### Postoperative Treatment

All patients were treated with sterile dressings, and plaster was used to fix the affected limb for 2 weeks. On the third day after the surgery, finger flexion and extension function exercises began, and after gypsum removal, wrist joint flexion and extension and rotation function exercises began.

### Efficiency Evaluation Indicators

#### Grip Strength and Wrist Joint Function

The recovery of grip strength, wrist flexion and extension, ulna‐radial deviation, and forearm rotation before the operation and 12 months after surgery were compared between the observation group and the control group (intragroup).

#### Length of Ulnar Variation

The length of ulnar variation was compared between the observation group and the control group 12 months after surgery (intergroup).

### Disabilities of the Arm, Shoulder, and Hand Score (DASH Score)

The DASH score is a questionnaire that assesses upper extremity function from the patient's perspective. The patient's self‐assessment was used to understand the degree of functional impairment of the affected limb. The DASH score was divided into two parts, containing 30 indicators. Each indicator corresponds to a score of five levels, namely, no difficulty (1 point), some difficulty (2 points), moderate difficulty but able to do (3 points), great difficulty (4 points), and unable to do (5 points). The DASH value was calculated by adding up the scores of 30 indicators and then calculating them according to the following formula: DASH value = (sum of scores of 30 indicators – 30 (lowest value))/1.20, so that the original score was transformed into 0–100 points, and the degree of upper limb function limitation was assessed according to the patient's score, where 0 points represented normal upper limb function and 100 points represented extremely limited upper limb function.

### Patient‐Rated Wrist Evaluation Score (PRWE Score)

The PRWE score is a patient self‐assessment questionnaire for wrist pain and mobility. A total of 15 items were included: five subitems related to pain, six subitems related to special activities, and four subitems related to daily activities. Each item was scored from 0 to 10. In the pain item, 0 points indicate no pain, and 10 points indicate the most pain. In the activity item, 0 points indicate no difficulty, and 10 points indicate a lot of difficulty. The score calculation method is as follows: the sum of the scores of the activity subitems was divided by 2, and the total score of the pain subitem was added to obtain a range of 0–100 points. The higher the score, the more severe the pain and dysfunction.

### Visual Analog Score (VAS Score)

The VAS score is one of the most commonly used pain scoring criteria. This was done by drawing a 10‐cm horizontal line on a piece of paper, with 0 at one end of the horizontal line indicating no pain, 10 at the other end indicating severe pain, and the middle part indicating different degrees of pain. Patients were asked to score their pain according to their pain conditions. The lower the score, the less severe the pain.

### Mayo Wrist Score

The Mayo wrist score was used to assess the recovery of wrist function in patients after surgery. The Mayo wrist score mainly includes four items: pain, function, grip strength, and range of motion (compared with the healthy side and affected hand only). The scores for each item were 0, 10, 20, and 25, and the scores were calculated by adding the scores together. A total score of <60 is considered to be poor, 60–80 is acceptable, 80–90 is good, and 90–100 is excellent.

### Statistical Analysis

SPSS 21.1 statistical software was used. Measurement data were expressed as means ± standard deviations. A *t* test of two independent samples was used for intergroup comparisons, and a paired *t* test was used for intragroup comparisons before and after comparisons. Grade data were expressed as counts, and the rank‐sum test was used. The difference was statistically significant (*p* < 0.05).

## Results

### Follow‐Up

**FIGURE 1:**
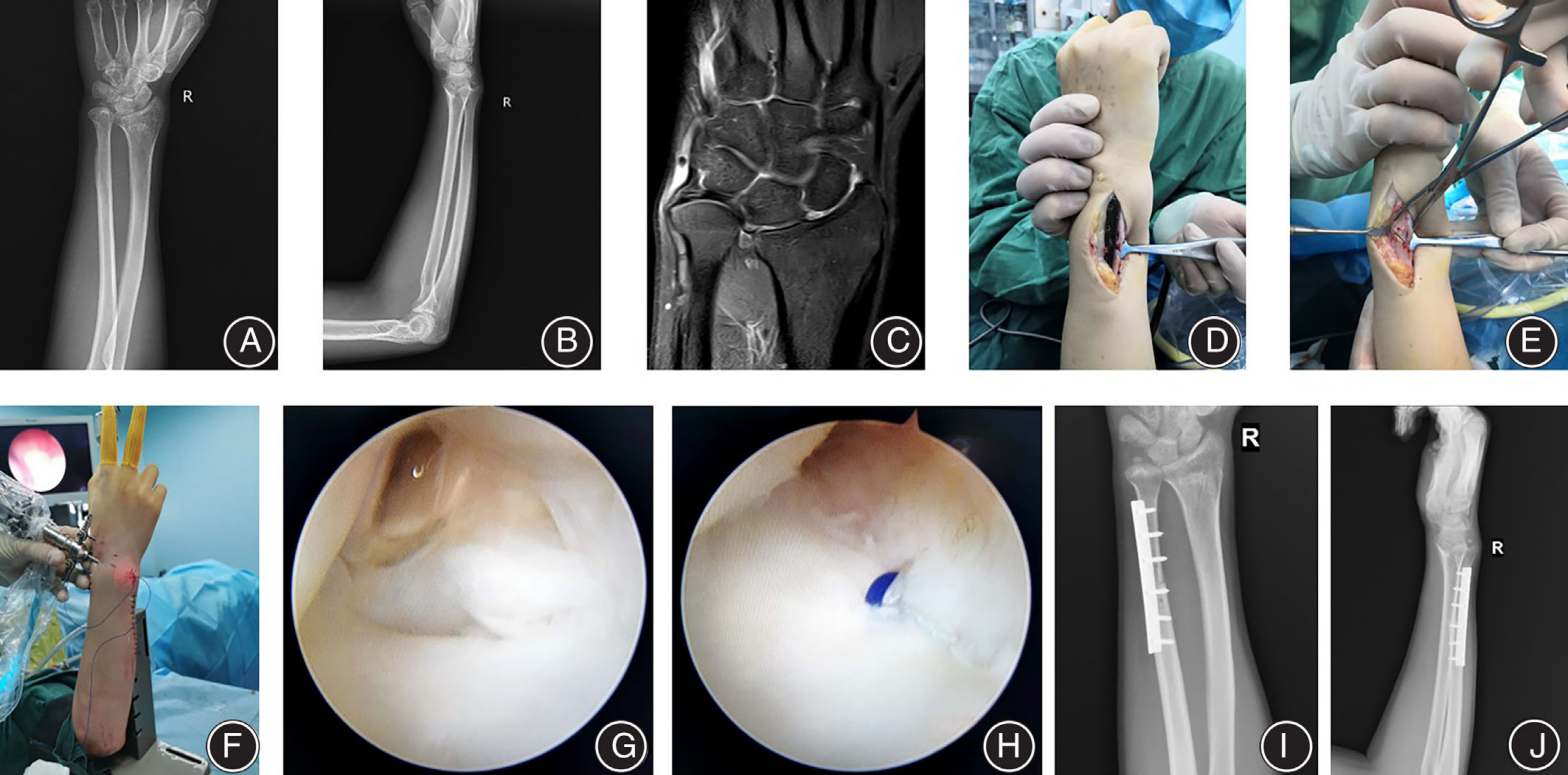
Typical case 1. Female, 36 years old, pain and limited mobility for 8 months due to long‐term activity in the right wrist joint, diagnosed with right wrist UIS, TFCC injury. Treated by wrist arthroscopy combined with oblique USO. (A, B) Preoperative anterolateral X‐ray films showed positive ulna variation; (C) Preoperative MRI showed positive ulnar variation combined with TFCC injury; (D, E) Intraoperative oblique USO. (F) Intraoperative arthroscopic exploration of the wrist; (G, H) Intraoperative suture of TFCC; (I, J) Postoperative X‐rays showed that the ulnar osteotomy was well‐aligned, and the ulnar positive variation had recovered

**FIGURE 2:**
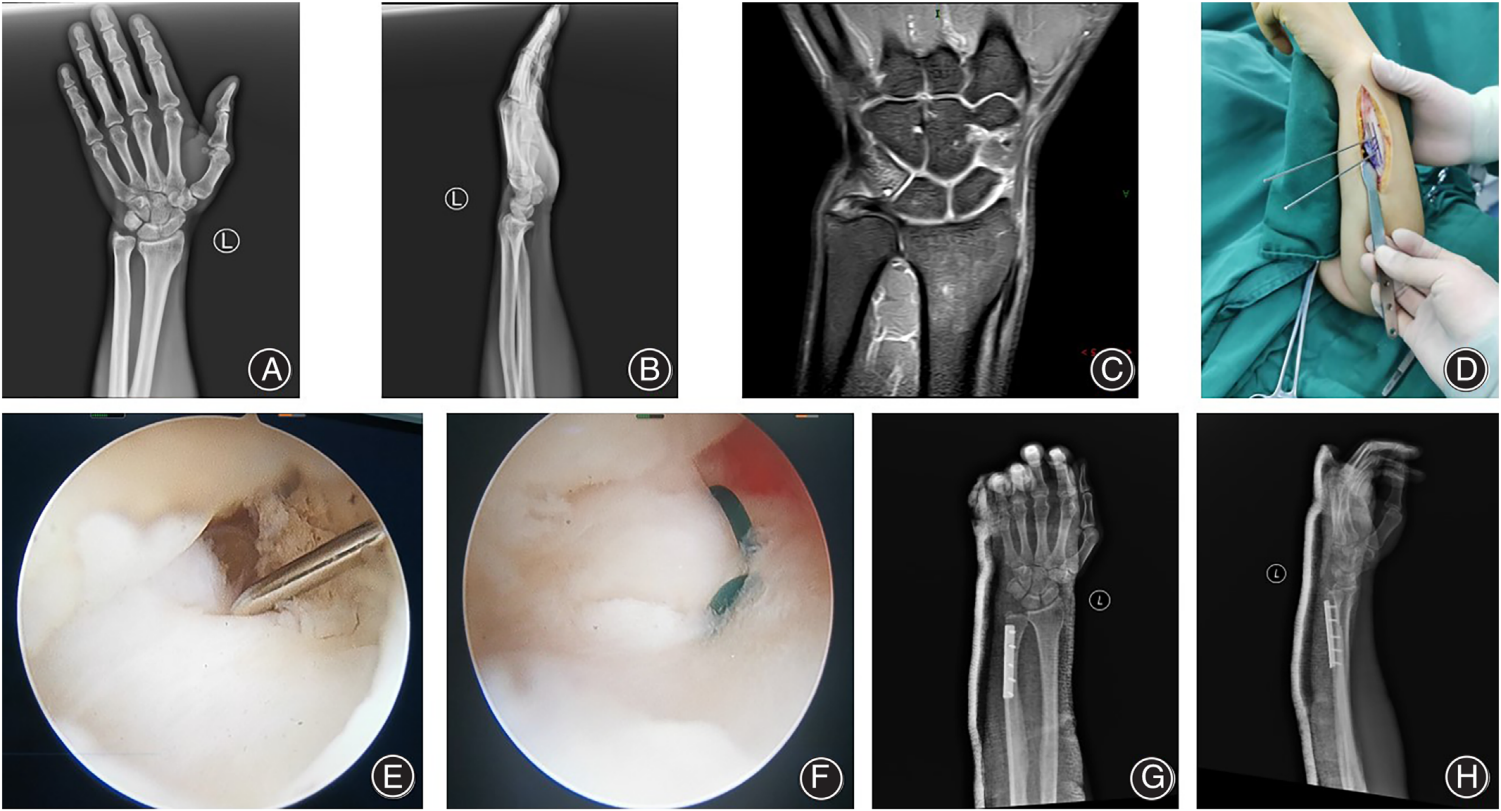
Typical Case 2. Male, 39 years old, with no obvious cause of left wrist pain and limited movement for 2 years, aggravated for 4 months, diagnosis of left wrist UIS, TFCC injury. Treated by wrist arthroscopy combined with oblique USO. (A, B) Preoperative anterolateral X‐ray films showed positive ulna variation; (C) Preoperative MRI showed positive ulnar variation combined with TFCC injury; (D) Intraoperative oblique USO. (E, F) Intraoperative suture of TFCC; (G, H) Postoperative X‐rays showed that the ulnar osteotomy was well‐aligned, and the ulnar positive variation had recovered

All patients were followed up for 12 to 36 months, with an average of 14.5 months. The osteounion was achieved in the oblique section of the ulna, with an average healing time of 13.6 weeks. Typical cases are shown in Figures 1 and 2.

### General Results

In the observation group, three cases of Palmer IIA, seven cases of Palmer IIB, 14 cases of Palmer IIC, and six cases of Palmer IID were confirmed by wrist arthroscopy. The observation group achieved ulnar shortening of 4.7 ± 1.0, and the control group achieved ulnar shortening of 4.9 ± 1.0. The difference was not statistically significant between the two groups (*p* = 0.467; Table 3).

### Clinical Improvement

At the 12‐month follow‐up, compared with before surgery, both groups' patients improved in grip strength, flexion‐extension, ulnar radial deflection, and forearm rotation, and the differences were statistically significant (*p* = 0.000; Tables 1 and 2).

**TABLE 1:** Comparison of grip strength, flexion and extension, ulnar radial deflection, and forearm rotation function in the observation group before and 12 months after surgery (mean ± SD)

	Grip strength	Flexion‐extension	Ulnar radial deflection	Forearm rotation
Before surgery	11.9 ± 3.2	108.9 ± 11.4	24.2 ± 6.1	129.7 ± 8.7
After surgery	26.4 ± 8.9	143.2 ± 9.5	37.3 ± 6.1	152.8 ± 12.4
*t* value	−9.356	−16.351	−11.295	−11.774
*p* Value	0.000	0.000	0.000	0.000

**TABLE 2:** Comparison of grip strength, flexion and extension, ulnar radial deflection, and forearm rotation function in the control group before and 12 months after surgery (mean ± SD)

	Grip strength	Flexion‐extension	Ulnar radial deflection	Forearm rotation
Before surgery	12.4 ± 2.9	108.1 ± 12.2	23.2 ± 5.5	126.6 ± 8.5
After surgery	22.3 ± 6.9	135.2 ± 11.0	32.5 ± 6.7	143.3 ± 11.8
*t* value	−8.674	−9.999	−7.756	−7.184
*p* Value	0.000	0.000	0.000	0.000

### Grip Strength

Before surgery, the average grip strength of the patient's affected limb in the observation group was 11.9 ± 3.2 kg, and at 12 months after surgery, it was 26.4 ± 8.9 kg; the difference was statistically significant (*p* = 0.000; Tables 1 and 2). In the control group, before surgery, the average grip strength of the patient's affected limb was 12.4 ± 2.9 kg, and at 12 months after surgery, it was 22.3 ± 6.9 kg; the difference was statistically significant (*p* = 0.000; Tables 1 and 2).

### Flexion‐Extension

Before surgery, the average flexion‐extension of the patient's affected limb in the observation group was 108.9 ± 11.4, and at 12 months after surgery, it was 143.2 ± 9.5; the difference was statistically significant (*p* = 0.000; Tables 1 and 2). In the control group, before surgery, the average flexion‐extension of the affected limb was 108.1 ± 12.2, and at 12 months after surgery, it was 135.2 ± 11.0; the difference was statistically significant (*p* = 0.000; Tables 1 and 2).

### Ulnar Radial Deflection

Before surgery, the average ulnar radial deflection of the patient's affected limb in the observation group was 24.2 ± 6.1, and at 12 months after surgery, it was 37.3 ± 6.1; the difference was statistically significant (*p* = 0.000; Tables 1 and 2). In the control group, before surgery, the average ulnar radial deflection of the patient's affected limb was 23.2 ± 5.5, and at 12 months after surgery, it was 32.5 ± 6.7; the difference was statistically significant (*p* = 0.000; Tables 1 and 2).

### Forearm Rotation

Before surgery, the average forearm rotation of the patient's affected limb in the observation group was 129.7 ± 8.7, and at 12 months after surgery, it was 152.8 ± 12.4; the difference was statistically significant (*p* = 0.000; Tables 1 and 2). In the control group, before surgery, the average forearm rotation of the affected limb was 126.6 ± 8.5, and at 12 months after surgery, it was 143.3 ± 11.8; the difference was statistically significant (*p* = 0.000; Tables 1 and 2).

### Functional Evaluation

At 12 months after surgery, the observation group's DASH score, PRWE score, and VAS score were better than those of the control group (*p* = 0.000; Table 3), the difference was statistically significant. At the last follow‐up, the excellent and good rates of the Mayo wrist score observation group were higher than those of the control group, and the difference between the two groups was statistically significant (*p* = 0.032; Table 4).

**TABLE 3:** Comparison of DASH score, PRWE score, VAS score, and ulnar variation between the two groups 12 months after surgery (mean ± SD)

Groups	DASH score	PRWE score	VAS score	Ulnar variation
Observation	14.4 ± 6.1	15.0 ± 3.8	1.4 ± 0.7	4.7 ± 1.0
Control	21.7 ± 4.5	22.3 ± 5.5	2.5 ± 1.1	4.9 ± 1.0
*t* value	−5.279	−6.030	−4.761	−0.732
*p* Value	0.000	0.000	0.000	0.467

**TABLE 4:** Comparison of excellent and good rates between the two groups 12 months after surgery

Groups	Mayo wrist score				
	Excellent	Good	Can	Bad	Excellent and good rates
Observation	25	3	2	0	93.3
Control	17	9	4	0	87.5
*Z* value					−2.149
*p* Value					0.032

### DASH Score

At 12 months after surgery, the observation group's DASH score was 14.4 ± 6.1, and the control group was 21.7 ± 4.5. The difference between the two groups was statistically significant (*p* = 0.000; Table 3).

### PRWE Score

At 12 months after surgery, the PRWE score of the observation group was 15.0 ± 3.8, and that of the control group was 22.3 ± 5.5. The difference between the two groups was statistically significant (*p* = 0.000; Table 3).

### VAS Score

At 12 months after surgery, the VAS score of the observation group was 1.4 ± 0.7, and that of the control group was 2.5 ± 1.1. The difference between the two groups was statistically significant (*p* = 0.000; Table 3).

### Excellent and Good Rates of Mayo Wrist Scores

At the last follow‐up, the observation group's excellent and good rates of Mayo wrist joint scores were 93.3%, and those of the control group were 87.5%. The difference between the two groups was statistically significant (*p* = 0.032; Table 4).

### Complications

One patient in the control group developed postoperative palsy of the dorsal carpal branch of the ulnar nerve, which was not specifically treated and returned to normal on its own after 2 months.

## Discussion

### Analysis of Research Results

The results of this study show that both groups of patients can effectively relieve pain and restore grip strength and wrist range of motion after oblique USO, proving that oblique USO is a good method for the treatment of UIS. The observation group outperformed the control group in terms of DASH score, PRWE score, and VAS score, and the Mayo wrist score at the last follow‐up also showed that the observation excellent and good rates were better than those of the control group. We believe the reason for this is that the use of the wrist arthroscopy technique provides better pain relief to the patients and helps them to perform early functional exercises, which in turn leads to better restoration of joint function. The use of the wrist arthroscopy technique enables the exploration, cleaning, and repair of soft tissues such as the TFCC, which is an important component of the ulnar side of the wrist and is the main structure that maintains the stability of the ulnar side of the wrist, and its injury can cause varying degrees of pain and restricted movement of the wrist joint^18^. Palmer and Werner^19^ classified TFCC injuries caused by UIS into five types, namely, IIA, B, C, D, and E, from mild to severe, with each type being treated accordingly by cleaning the TFCC and suturing it separately. In the past, conservative treatment or open repair was used, the treatment effect was general, and the wound was large. In recent years, with the development of wrist arthroscopy technology, it can not only directly examine the wrist joint but is also the “gold standard” for UIS diagnosis^20^. In addition, it can clean the proliferated synovial tissue and free articular cartilage in a minimally invasive manner under the microscope to repair TFCC^21^. Some scholars believe that because of the positive correlation between UIS and TFCC degeneration and chondromalacia of the lunate and triquetrum, wrist arthroscopy should be the main method for the treatment of UIS^22^. Some scholars believe that the combination of wrist arthroscopy and ulnar osteotomy is an ideal treatment for unstable UIS with TFCC injury^23^, ^24^. Roh *et al*.^25^ divided 44 patients with UIS into a simple USO group and a USO combined with arthroscopic debridement group. The results showed that the pain score in the USO combined with arthroscopic debridement group was better than that in the USO alone group at the third month of follow‐up, which was similar to the results of our study.

### Advantages of Wrist Arthroscopy Combined with Oblique USO

(i) Oblique osteotomy can achieve ulnar neutral or negative variation, reduce the pressure of ulna on the wrist, and tighten TFCC to stabilize the lower radioulnar joint, and the study confirmed that oblique osteotomy can obtain a shorter healing time and lower complication rate than transverse osteotomy^26^; (ii) Because the ulna will change in the process of rotation, for some patients whose ulna positive variation is not obvious on orthopaedic X‐ray film, wrist arthroscopy can directly observe the ulna variation in the process of rotation to make a clear diagnosis of the disease and the degree of injury and then choose the best operation plan^27^; (iii) Wrist arthroscopy can clean up the proliferative synovial tissue and free articular cartilage and repair the TFCC injury to effectively improve the symptoms and restore wrist function; (iv) Wrist arthroscopy has the advantage of being minimally invasive, which can ensure minimal injury in the joint at the time of treatment, is helpful for the rehabilitation of patients, and has the advantages of a low complication rate and quick postoperative recovery^22^.

### Key Points of Surgery

(i) Ulnar osteotomy should not be too close to the distal end of the ulna, since it can easily damage the dorsal carpal branch of the ulnar nerve. (ii) The length of ulnar osteotomy should be accurately designed before ulnar osteotomy. The ideal range of ulnar variance is 1–2 mm longer than the positive ulnar variance so that the ulna can reach neutral variance or negative variance of 1–2 mm. This will affect the outcome of ulnar shortening osteotomy. If the osteotomy is too short, it will cause the ulnar impact to still exist, thus failing to obtain satisfactory results. If the osteotomy is too long, the bone will be prone to delay or nonunion and complexity, regional pain syndrome, and so on. (iii) When repairing TFCC perforation under arthroscopy, attention should be given to avoid damaging the ulnar attachment of TFCC and causing instability of the distal radioulnar joint. (iv) Arthroscopy is an invasive examination, but the wrist joint space is small, and the structure is complex. Therefore, accurate positioning should be made in the approach to avoid injury to nerves, blood vessels, and other soft tissues.

### Limitations

This study has several limitations. (i) The sample size included in this study is relatively small, which may lead to deviation in the research results. In the future, it is necessary to conduct prospective randomized controlled trials with larger samples to improve the level of evidence. (ii) All patients in this study were followed up for a short time and lacked long‐term wrist function comparison. Therefore, further follow‐up is still needed to determine the efficacy. (iii) The use of wrist arthroscopy may lead to greater treatment costs for patients. With the gradual popularization of this technology in the future, its price is expected to be further reduced.

## Conclusion

Both oblique USO alone and combined wrist arthroscopy can effectively relieve the symptoms of UIS and restore wrist joint function. Compared with oblique USO alone, combined wrist arthroscopy can better reduce the pain of patients, restore wrist function, and improve excellent and good rates. If the economic conditions of the patients permit, it is clinically worthy of promotion.

## Author Contribution

Prof. Xiaohui Wang and Dr. Hailong Zhang mainly completed the operation process. Dr. Laifu Zhang and Dr. Dong Jiahe collected data. Dr. Chengyin Lu and Dr. Pengtao Wang wrote the first draft of the manuscript. Dr. Lei Yang revised the manuscript. This work was guided by Prof. Xiaohui Wang and Prof. Hui Xiong.

## Ethics Statement

All authors listed meet the authorship criteria according to the latest guidelines of the International Committee of Medical Journal Editors and all authors are in agreement with the manuscript.

## Conflict of Interest

The authors declare no conflict of interest. No benefits in any form have been, or will be, received from a commercial party related directly or indirectly to the subject of this article.
